# Auxological and endocrine findings in narcolepsy type 1: seventeen-year follow-up from a pediatric endocrinology center

**DOI:** 10.3389/fendo.2023.1037398

**Published:** 2023-06-15

**Authors:** Sara Casale, Valentina Assirelli, Fabio Pizza, Antonio Balsamo, Monia Gennari, Andrea Pession, Giuseppe Plazzi, Alessandra Cassio

**Affiliations:** ^1^ Specialty School of Pediatrics, Alma Mater Studiorum, University of Bologna, Bologna, Italy; ^2^ Department of Biomedical and Neuromotor Sciences, University of Bologna, Bologna, Italy; ^3^ Istituto di Ricovero e Cura a Carattere Scientifico (IRCCS) Istituto delle Scienze Neurologiche di Bologna, Bologna, Italy; ^4^ Department of Medical and Surgical Sciences, University of Bologna, Bologna, Italy; ^5^ Pediatric Unit, Istituto di Ricovero e Cura a Carattere Scientifico (IRCCS) Azienda Ospedaliero-Universitaria di Bologna, Bologna, Italy; ^6^ Department of Medical and Surgical Sciences, Istituto di Ricovero e Cura a Carattere Scientifico (IRCCS), University of Bologna, Bologna, Italy; ^7^ Department of Biomedical, Metabolic and Neural Sciences, University of Modena and Reggio-Emilia, Modena, Italy

**Keywords:** narcolepsy type 1, obesity, central precocious puberty, final height, sodium oxybate

## Abstract

**Introduction:**

Narcolepsy Type 1 (NT1) is a rare hypersomnia of central origin linked to hypocretin deficiency, most frequently arising at pediatric age. NT1 could be associated with endocrine comorbidities involving the neuroendocrine axis, predominantly obesity, and Central Precocious Puberty (CPP). The primary aim of this study is the evaluation of endocrine and auxological parameters at diagnosis and during follow-up in patients with NT1, treated with Sodium Oxybate (SO) or not.

**Methods:**

We retrospectively evaluated the auxological, biochemical, and radiological parameters of 112 patients referred to our Center between 2004-2022. The design of our study is cross-sectional at the time of diagnosis followed by a longitudinal follow-up.

**Results:**

Our study confirms an increased frequency of CPP and obesity in patients with NT1. At first evaluation, obesity was found in 31.3% of patients, while overweight was found in 25.0%. A diagnosis of CPP was made in 19.6% of patients. Interestingly, this group showed a significantly lower level of CSF-hypocretin (hrct-1) at diagnosis compared to others. We found an improvement in BMI SDS in the SO-treated group compared to untreated patients, and this trend persisted also at 36 months of follow-up (0.0 ± 1.3 vs 1.3 ± 0.4; p<0.03). Sixty-three patients reached their final height, with a median SDS of 0.6 ± 1.1 in boys and 0.2 ± 1.2 in girls.

**Discussion:**

To our knowledge, these are the first results regarding the final height in a large series of pediatric patients with NT1, with a normal range of IGF1-SDS levels and stature SDS.

## Introduction

Narcolepsy type 1 (NT1) is a rare sleep disorder, clinically characterized by the presence of Excessive Daytime Sleepiness (EDS) and cataplexy (1–3). NT1 seems to be attributable to an immune-mediated destruction of hypocretin (or orexin) producing neurons in genetically predisposed subjects. The loss of the orexin system has been associated with alterations in sleep and the neuroendocrine system, causing modification in energy balance, eating behavior, glucose metabolism, and modulation of the hypothalamic pituitary axis (4).

Despite major advances in understanding the mechanisms underlying NT1 pathophysiology, treatment options remain still symptomatic (1, 2). In fact, treatment choices are not influenced by metabolic characteristics but only by neurological ones. The actual treatment options are SO, Pitolisant, Modafinil, and Venlafaxina, alone or in combination. Furthermore, only SO has proved to be safe and effective in children and adolescents suffering from NT1 (5, 6). SO is the sodium salt of gamma-hydroxybutyric acid that has a complex and not entirely known stimulating action of GABA-B receptors (5, 6). Since 2020, SO is registered in Europe and the United States to treat EDS and cataplexy in children older than 7 years old with narcolepsy. Recently, a study demonstrated the efficacy and safety of the use of Pitolisant in pediatric patients with NT1 (7).

Regarding endocrinological comorbidity, NT1 in children seems to be associated with obesity and Central Precocious Puberty (CPP) (8, 9). Recently, a case report described also a possible association between NT1 and Growth Hormone (GH) deficiency (10). To our knowledge, the effect of NT1 and its treatment on the growth and final height of patients remains still unknown.

For the first time, Ponziani et al. (11) described the positive results of the treatment with SO on BMI of pediatric patients with NT1 during the first year of treatment (11). Although other studies have demonstrated the reduction of BMI in adult patients with NT1 receiving SO therapy (12), however the mechanism leading to weight loss is still unexplained, and no long-term follow-up studies have evaluated the effect of NT1 on anthropometric parameters, such as final height, or BMI (11).

The primary aim of this study is the evaluation of the endocrine-metabolic and anthropometric parameters at diagnosis and during follow-up in a cohort of one-hundred-and-twelve patients with NT1 referred to our Pediatric Endocrinology Center. We focus on the final height and we evaluate the long-term effect of SO treatment in the endocrine-metabolic system. The secondary aim of this study is the identification of prognostic factors for endocrine complications in pediatric patients with NT1.

## Materials and methods

### Study participants and setting

We retrospectively evaluated the medical records of 112 consecutive patients (65 M and 47 F) referred to our Center for an endocrine evaluation in the context of NT1 from September 2004 to March 2022.

Inclusion criteria were the following: diagnosis of NT1 performed at the Narcolepsy Center of the IRCSS Istituto delle Scienze Neurologiche di Bologna, Bologna, Italy; age between 2 and 18 years old. Exclusion criteria were diagnosis of NT2 and/or presence of other conditions or syndromes known to be associated with CPP and/or obesity.

### Study design and statistical analysis

The design of our study is cross-sectional at the time of diagnosis followed by a longitudinal follow-up. At diagnosis, we compared our 112 patients on the basis of having or not obesity/overweight and CPP. Non-parametric statistical between-group comparison were performed using Chi-Square test or Student T test for categorical or continuous variables.

During the follow-up, we longitudinally collected anthropometric parameters of patients with NT1, such as height, BMI, and pharmacological treatment. BMI SDS was evaluated as mean and standard deviation (SD) and patients were compared based on the baseline treatment with SO or not. Finally, the first visits’ BMI SDS was compared to the last visits’ BMI SDS and separately compared based on SO treatment at baseline.

In total, 63 patients (30 M and 33 F) reached their final height (FH) and these were compared to their respective target height. Student T test was used to compare BMI SDS between groups at different follow-up periods, and to compare the final and target height of the 63 patients, as well as in the subgroup of patients with and without SO treatment (50 patients, 23 M and 27 F) and with and without CPP (15 patients, 5 M, and 10 F).

Continuous and categorical data were examined with descriptive statistics (mean ± standard deviation and range) and frequency in the entire population and patient subgroups respectively. Statistical comparisons were assessed by Student T or chi-square tests for continuous or categorical data, respectively. Multivariate linear repeated measures analyses, including CPP and baseline ESS as covariates, were performed to confirm the effect of SO treatment on BMI z-score change adjusting for baseline features. A p-value < 0.05 was considered statistically significant.

### Narcolepsy type 1 diagnosis

Diagnosis of NT1 was performed by submitting all 112 patients to polysomnography (PSG) followed by a Multiple Sleep Latency Test (MLST) with five nap opportunities, and a blood sample for HLA DQB1 * 06: 02 genotyping (2).

A cephalorachidian sample to determine CSF hrct-1 was available in 100 patients. CSF hrct-1 levels were assessed by radioimmunoassay using the Stanford reference sample.

To assess daytime sleepiness, a modified ESS (Epworth Sleepiness Scale- CHAD-ESS) was administered to one-hundred-and-twelve patients. A score greater than 11/24 was considered pathological (13).

### Endocrine and anthropometric evaluations

For all 112 patients, we retrospectively investigated from medical records familiarity for precocious puberty, hypertension, hypercholesterolemia, obesity, diabetes, and other endocrine disorders.

The auxological parameters such as height, weight, and BMI were compared by sex and age using the Italian growth curves in order to determine the standard deviation (SD) and percentiles (14). The height was measured using a Harpenden stadiometer and expressed as SD (15). The target height was calculated based on the gender-corrected mean height of the parents (15, 16). Final stature was defined as growth <0.3 cm over a period of more than 6 months associated with complete or almost complete sealing of the conjugating cartilages (17). The BMI was calculated according to the formula: weight (kg)/height (m2). The determination of BMI percentile was used to define patients as normal weight, overweight and obese, in accordance with recent Italian consensus criteria: a BMI value below the 85th percentile defines a normal weight, a BMI value between 85-97st percentile defines a condition of being overweight and a BMI above 97th percentile defines a condition of obesity (18).

Systolic and diastolic blood pressure (BP) values were measured in supine position. The classification approach of BP in children includes normal, prehypertension, stage I hypertension, and stage II hypertension, according to normative percentiles of BP averaged over 3 occasions. We defined hypertension if the measurements were > 90th percentile (18).

Pubertal development was assessed according to Tanner and Whitehouse’s criteria (19, 20).

Blood samples were collected in fasting conditions between 07.00 and 09.00 in the morning. The following baseline hormonal and metabolic parameters were evaluated: TSH, free T3, free T4, ACTH, cortisol, free urinary cortisol, prolactin, IGF1, estradiol in girls, testosterone in boys, fasting glucose, insulin, total cholesterol, LDL cholesterol, HDL cholesterol, triglycerides. In the case of suspected CPP, the following examinations were also performed: bone age (BA), pelvic ultrasound in girls, and dynamic test with GnRH. BA was evaluated using the Greulich and Pyle Atlas (21). The conventional GnRH stimulus test was performed by intravenous administration of 50-100 μg of the drug, with samples taken at 0’, 30’, and 60’ (19).

Pelvic ultrasound was performed trans-abdominally by a group of radiologists’ experts in pediatric evaluations using a Convex echotomography with B-mode ultrasound signal and wavelength varying between 3.5 and 5.0 MHz (Philips). The subject was evaluated in a supine position and with an extended bladder, so uterine measurements (longitudinal diameter (DL), transverse diameter (DT) and anteroposterior diameter (DAP)), ovarian measurements and visibility of the endometrial rhyme were carried out. The uterine and ovarian volumes were obtained through the elyxoid formula (DL x DT x DAP x 0.52) (19, 22).

The diagnosis of idiopathic CPP was made following the Consensus criteria (23, 24):

- appearance of signs of pubertal development before the age of 8 years in females and 9 years in males,
- serum levels of luteinizing hormone (LH) > 5 mIU/mL after GnRH dynamic test,
- ultrasound data: uterine longitudinal diameter (ULD) > 36 mm, body/neck ratio > 1 or presence of endometrial rhyme
- normal Magnetic Resonance Imaging (MRI) of the hypothalamus-pituitary region.

The Oral Glucose Tolerance Test (OGTT) was performed through the administration of 1.75 g glucose/kg of ideal weight, with a maximum dose of 75 g, and subsequent blood samples were taken at baseline and after 30, 60, 90, and 120 minutes from the glucose load. We defined Impaired Fasting Glycaemia (IFG) as fasting plasma glucose between 100 mg/dl and 125 mg/dl and Impaired Glucose Tolerance (IGT) as plasma glucose between 140 mg/dl and 199 mg/dl at 120 min. Type 2 Diabetes (T2D) was defined as fasting plasma glucose > 126 mg/dl and plasma glucose at 120 min ≥ 200 mg/dl (25, 26).

## Results

### Clinical, metabolic, hormonal, and anthropometric parameters detected at diagnosis of NT1

A total of 112 patients fulfilled the inclusion criteria and were recruited for the study: 65 boys and 47 girls. The mean age at onset of symptoms was 8.4 ± 2.4 years, while the mean age at diagnosis was 10.1 ± 3.1 years. HLA DQB1*06:02 positivity was found in 96.2% of subjects. The mean level of hypocretin was 17.7 ± 25.9 [0.0 – 109.0], with 93.3% of patients showing a level of hypocretin < 100 pg/mL. In 47 patients the hypocretin level was undetectable. Mean hypocretin levels at diagnosis were significantly lower in the group of patients with CPP compared to the group without CPP (15.7 ± 17.8 vs 19.1 ± 27.6) (p<0.01). On the other hand, we did not find statistically significant differences in hypocretin levels when comparing obese/overweight patients to others (19.1 ± 23.5 vs 17.5 ± 29.3) (p>0.05).

A diagnosis of CPP after the diagnosis of NT1 was found in 19.6% (22/112). The frequency of CPP was 15.4% (10/65) in males and 25.5% (12/47) in females.

For 12 subjects we started treatment with a GnRH analogue according to the guidelines (23). Only in one case, there was familiarity for CPP. All hormonal and metabolic parameters in these patients were in the normal range.

Through an analysis conducted with descriptive statistics, we found a significantly lower age at NT1 onset in those with CPP compared to those without this comorbidity (p<0.03). Clinical and anthropometric parameters detected at diagnosis in patients with NT1 with and without CPP are reported in **Table 1**.

**Table 1:** Clinical and anthropometric parameters detected at diagnosis in patients with NT1.

	NT1 (112)	With CPP (22/112)	Without CPP (90/112)	P value	With obesity/ overweight (63/112)	Without obesity/ overweight (49/112)	P value
Age at onset, years	8.4 ± 2.4	7.1 ± 2.0 †	8.7 ± 2.4	<0.01	8.3 ± 2.5	8.5 ± 2.3	n.s.
Age at diagnosis of NT1, years	10.1 ± 3.1	8.8 ± 2.4 $	10.4 ± 3.2	<0.03	10.3 ± 3.2	10.0 ± 3.0	n.s.
Height at diagnosis, SDS	0.5 ± 1.0	0.9 ± 1.2	0.4 ± 1.0	n.s.	0.7 ± 1.0	0.4 ± 1.1	n.s.
BMI at diagnosis, SDS	1.0 ± 1.1	1.1 ± 0.8	1.0 ± 1.2	n.s.	1.8 ± 0.6 *	0.0 ± 0.8	<0.01
Fasting plasma glucose, mg/dL	82.1 ± 6.0	79.4 ± 6.7	79.7 ± 9.0	n.s.	82.9 ± 6.2	80.6 ± 5.3	n.s.
Fasting plasma insulin, µIU/mL	11.1 ± 6.4	11.2 ± 5.3	11.3 ± 6.9	n.s.	12.3 ± 7.5	10.0 ± 4.6	n.s.
Systolic and Diastolic Pressure > 90, %	20.0	4.9 †	22.0	<0.01	25.6 *	1.2	<0.01
HDL cholesterol, mg/dL	54.7 ± 14.1	51.4 ± 12.9	55.5 ± 14.4	n.s.	52.0 ± 13.2 §	59.0 ± 14.9	<0.02

†p < 0.01 between NT1 and CPP and NT1 without CPP, $ p < 0.03 between NT1 and CPP and NT1 without CPP. *p < 0.01 between NT1 with obesity/overweight and NT1 without obesity/overweight. §p < 0.02 between NT1 and obesity/overweight and NT1 without obesity/overweight. Statistical comparisons were assessed by Student T or chi-square tests for continuous or categorical data, respectively. A p-value < 0.05 was considered statistically significant. n.s., Not Significant.

Excluding patients with CPP, at diagnosis 63.3% of subjects were already in the pubertal stage, while 36.7% were prepubertal. In prepubertal males (27/55), a testicular volume > 4 mL appeared at an average age of 10.8 ± 1.1 [9.0 – 13.5]. In prepubertal females (6/35), thelarche appeared at an average age of 9.4 ± 0.6 [8.1 – 10.5]. The average age at menarche was 11.3 ± 1.2 [9.0 – 15.0] years.

Obesity was found in 31.3%, while overweight in 25.0%, without significant differences between males and females. No patient at the time of diagnosis had a height lower than -2 SD. Hormonal and metabolic parameters at diagnosis were within the normal range also in these patients. Using descriptive statistics and Student’s T-test we found no statistically significant differences between NT1 patients with obesity/overweight and NT1 patients without obesity/overweight when analyzing blood glucose and insulin levels, while we found lower HDL-cholesterol values in the obesity/overweight group (59.0 ± 14.9 vs 52.0 ± 13.2) (p<0.02).

At diagnosis, we found blood pressure (BP) > 90% more frequent in those who were overweight/obese than in those without this comorbidity (25.6% vs 1.2%) (p<0.01). The clinical, metabolic, hormonal, and anthropometric parameters detected at diagnosis in patients with NT1 with and without obesity/overweight are shown in **Table 1**.

### Clinical and anthropometric parameters at follow-up

Our population included 112 patients. In our long follow-up, with a median time of 30.8 ± 23.7 [2.9 – 93.8] months, and we evaluated the effects of SO treatment on weight loss in pediatric patients with NT1 and their final height. For the purpose of the study, we analyzed the different treatments of our patients at the time of diagnosis: 18 untreated and 94 treated. The therapeutic approach after diagnostic evaluation and at follow-up has been driven by predominant symptoms (daytime sleepiness, cataplexy, disturbed nocturnal sleep, and their combination), by illness severity, and by an active discussion with patients’ parents. We preferred to start with a monotherapy, preferably using active drugs on different NT1 symptoms, such as SO or Pitolisant, adding drugs according to clinical judgement, evaluating the patients during the follow-up also from a neurological point of view and always involving the parents on the clinical choice, thus always preferring a patient-centered treatment (27).

At the first evaluation 64/112 patients have undergone treatment with SO, and the specific treatments our patients underwent are described in **Table 2**.

**Table 2:** The specific treatment for each patient group during each time of the follow-up of this study.

Specific treatment		Patients treated at T0	Patients treated at T1	Patients treated at T2	Patients treated at T3
Sodium Oxybate (SO) alone		42	31	24	14
Pitolisant (P)	P alone	1	1	0	1
	P + SO	4	1	1	2
	P + M	0	1	2	0
	P + SO + M	0	0	1	0
Modafinil (M)	M alone	11	11	9	3
	M + SO	16	18	11	6
	M + V	4	2	2	0
	M + SO + V	2	1	2	0
Venlafaxina (V)	V + SO	0	1	1	0

T0, T1, T2 and T3 are the times at diagnosis, 12, 24 and 36 months of follow-up, respectively.

**Figure 1:**
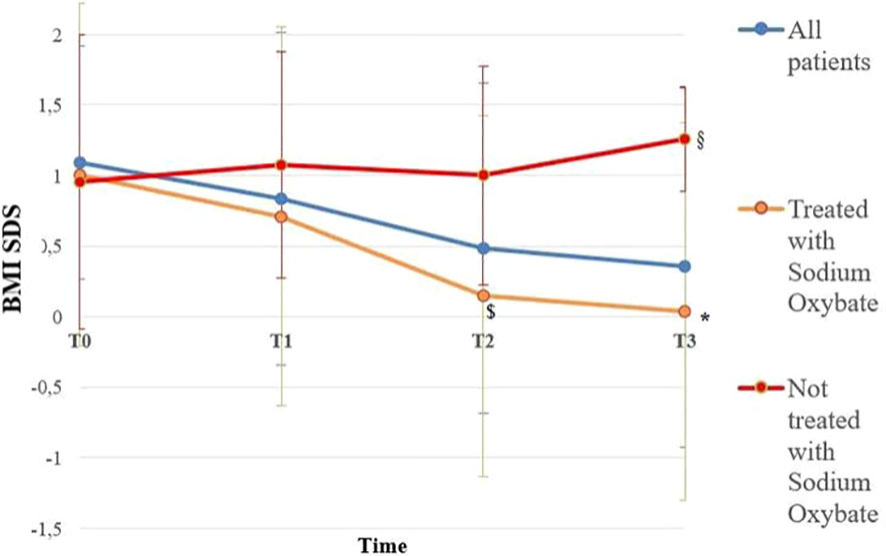
BMI SDS of all patients overall and divided according to Sodium Oxybate (SO) therapy at the baseline from the time of diagnosis up to 36 months. Blue line is representative of all patients with NT1 (112); orange line is representative of patients with NT1 treated with SO (64), red line is representative of Patients with NT1 not treated with SO (48). T0, T1, T2 and T3 are the times at diagnosis, 12, 24 and 36 months of follow-up, respectively. * p < 0.003 between BMI SDS in patients treated with SO at the baseline and BMI SDS at 36 months of follow-up, $ p < 0.007 between BMI SDS in patients treated with SO at baseline and patients not treated with SO at 24 months of follow-up, § p < 0.03 between BMI SDS in patients treated with SO at baseline and patients not treated with SO at 36 months of follow-up. Statistical comparisons were assessed by Student T for continuous data. A p value < 0.05 was considered statistically significant.

**Figure 2:**
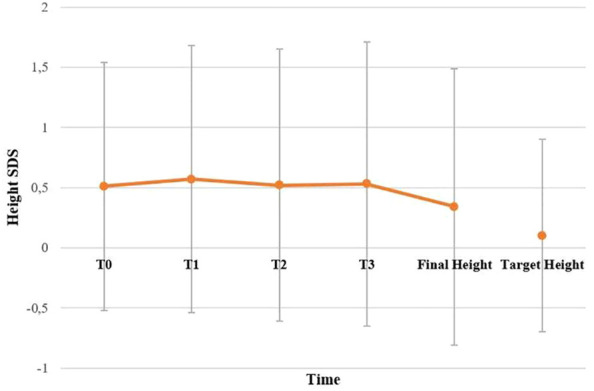
Height SDS of all patients with NT1 overall. T0, T1, T2 and T3 are the times at diagnosis, 12, 24 and 36 months of follow-up, respectively. No statistically significant difference has been found between final height compared to target height. Statistical comparisons were assessed by Student T for continuous data. A p value < 0.05 was considered statistically significant.

BMI SDS changes during follow-up are reported in **Table 3**. Patients who were treated with SO showed a significantly lower BMI SDS than those who were not treated with SO at baseline and compared with the BMI SDS at the time of diagnosis. The SO effect was maintained during the 36-months of follow-up. A graphic representation of the BMI SDS at diagnosis and during follow-up of all patients overall and divided according to SO therapy is reported in **Figure 1**. Multivariate repeated measures analysis on BMI SDS change in patients with and without SO treatment, confirmed a significant effect of SO (p = 0.007), despite the inclusion of baseline clinical covariates (CPP and ESS with a p-value of 0.066 and of 0.395 respectively). A graphic representation of the height SDS of all patients with NT1 overall is reported in **Figure 2**. All height SDS were greater than -2 SD and in the target height range. Dividing NT1 patients with and without CPP and with and without obesity/overweight, there were no significant differences between groups in height SDS. Mean IGF1-SDS levels were 0.4 ± 1.0 (296.8 ± 130.4 ug/L). Sixty-three patients (30 M, 33 F) reached their final height. Height SDS was 0.6 ± 1.1 in boys and 0.2 ± 1.2 in girls. Target parental height was 0.1 ± 0.8 SDS in boys and 0.3 ± 0.7 SDS in girls.

**Table 3:** BMI SDS of patients with NT1 divided according to Sodium Oxybate (SO) therapy at the baseline from the time of diagnosis up to 36 months.

BMI SDS	T0 (n.)	T1 (n.)	T2 (n.)	T3 (n.)
Patients with NT1 treated with SO	1.0 ± 1.2 $ (64)	0.7 ± 1.3 (50)	0.2 ± 1.3 * (34)	0.0 ± 1.3 § (20)
Patients with NT1 not treated with SO	1.0 ± 1.0 (48)	1.1 ± 0.8 (27)	1.0 ± 0.8 (23)	1.3 ± 0.4 (7)

T0, T1, T2 and T3 are the times at diagnosis, 12, 24 and 36 months of follow-up, respectively. *p < 0.007 between BMI SDS in patients treated with SO at baseline and patients not treated with SO at 24 months of follow-up, §p < 0.03 between BMI SDS in patients treated with SO at baseline and patients not treated with SO at 36 months of follow-up, $p < 0.003 between BMI SDS in patients treated with SO at the baseline and BMI SDS at 36 months of follow-up. (n.) Numbers of subject for each group. Statistical comparisons were assessed by Student T for continuous data. A p value < 0.05 was considered statistically significant.

## Discussion

The primary aim of this study was the evaluation of the endocrine-metabolic and auxological parameters at diagnosis and during follow-up in a cohort of 112 subjects with NT1, followed at our Pediatric Endocrinology Center. To our knowledge, this is the first study evaluating the endocrine-metabolic aspects in pediatric patients with NT1 with a long-term follow-up of 30.8 ± 23.7 months [2.9 – 93.9] and with an important number of patients that had reached the final height (63 subjects).

Our study confirms an increased frequency of obesity in patients with NT1 compared to the general pediatric population in Italy (https://www.epicentro.iss.it/okkioallasalute/indagine-2019-dati). Obesity was found in 31.3%, while overweight in 25.0%, without significant differences between males and females. As previously described (28, 29), overweight and obese subjects presented significative metabolic alterations compared to other patients with NT1, such as a significative reduction in HDL cholesterol (p<0.02) and the detection of a blood pressure in the upper limits (p<0.01). A possible explanation could be due to the lack of hypocretin, which seems involved in the maintenance of energy homeostasis (30). The study of Hara et al., in fact, demonstrates a reduction in basal metabolic rate in transgenic mice (31, 32). Another explanation could be due to the reduced hypothalamic binding of leptin, subsequently to the reduction of orexigenic neurons and so of the leptin receptors in them, which results in hyperphagia and impaired energy homeostasis (33).

In our study, we also confirm a higher frequency of CPP in patients with NT1 than reported in the general population (19.6% vs 0.2% in females and 0.05% in males) (24), with an equivalent gender distribution between males and females that distinguish these subjects from the cohort of idiopathic CPP (34), indeed, although we found a lower frequency of CPP in male patients with NT1 than in females, this difference was not significant (p>0.05). In our opinion, this in part may result from the greater diagnostic timeliness resulting from the regular follow-up these patients undergo, but it could also express an overriding effect of neurotransmitter deficiency at the level of the hypothalamic-pituitary axis. Recent studies suggest hypocretin’s role as the main actor of this comorbidity, as it directly affects GnRH (Gonadotropin Releasing Hormone) secretion, whose role is crucial in determining the onset of puberty (34). Interestingly, we found a significant reduction of the hypocretin levels in the CSF of these patients, compared to the other NT1 patients (p<0.01). As suggested by the literature review conducted by Melzi et al. (35), although previous studies are not currently exhaustive, we could speculate that CSF-hypocretin levels at diagnosis could be an important predictive factor leading to a rapid evaluation of this endocrine comorbidity. Orexin acts at different levels of the hypothalamic pituitary axis. Several studies suggest that orexin may act directly on GnRH neurons modulating their electrical activity, GnRH synthesis, and release. The absence of orexin in NT1 could favor the early activation of GnRH-secreting neurons and therefore the onset of puberty (35).

A limit of this evidence is that 63.3% of patients, excluding the CPP group, were already pubertal at diagnosis. This observation and the finding that, excepted CPP patients, the medium age at pubertal onset or menarche was on average one year earlier than in the general Italian population (36), suggest an effective causal role of NT1 in this phenomenon. These conditions hypothesize a possible synergistic effect between the hypocretinergic system and the regulation of the hypothalamic pituitary axis (34).

Ponziani et al. (11) described for the first time the positive effects of treatment with SO on BMI SDS in the first year of treatment (11), while the mechanism leading to weight loss are still unexplained. Either the recovery of slow-waves sleep, a more consolidated sleep–wake cycle in the 24 h and its beneficial consequences on daily activities, or the sleep-driven reconstruction of a circadian GH secretion with improved lipolysis process or the enhancement in sympathetic and cardiovascular functions could be considered implicated in SO-induced weight decrease. Furthermore, Ponziani et al. (37) showed that only SO improves weight, alone or in combinate-treatment, in the first year of use, after preliminary work showing a significant impact of SO treatment on weight only in patients under SO monotherapy (11). However, no long-term follow-up studies have evaluated the effect of NT1 on anthropometric parameters, such as final height, or BMI (11). We analyze BMI SDS of 112 NT1 patients in order to evaluate the effect of SO over time and we find that the favorable effect of therapy on BMI SDS persists beyond the first year of treatment in patients receiving monotherapy or in combination therapy with SO. In fact, after 36 months of follow-up, those who underwent SO therapy show reduced BMI SDS values ​​compared to patients not on SO therapy.

However, only large retrospective epidemiological studies can demonstrate the association between SO treatment and BMI SDS, without proving causality. Despite treatment choices being decided on a clinical basis focused on the relevance of neurological symptoms (i.e., EDS and cataplexy), and shared with active discussion with parents, a selection bias could not be excluded in the absence of a randomized approach. The long-term stabilization of BMI associated with SO treatment allows speculation on the central role of sleep on body weight control in patients with NT1.

A possible negative effect of NT1 has also been hypothesized on GH secretion and therefore on the growth trend of NT1 pediatric patients (29). To our knowledge, these are the first results regarding the final height in a large series of pediatric patients with NT1 (63 subjects reached their final height). Differently from the previous work (11) in which a GH deficiency was found in patients with NT1, our work does not seem to confirm the hypothesis of GH deficiency in patients with NT1. About the possible negative effect on final height, our evidence appears to be reliable for the number of subjects in which we tested GH and is confirmed also by normal IGF1 levels in our patients. In fact, we did not find significant differences between the final height of patients with NT1 and their target height, and this is also confirmed in patients with CPP, without gender differences. The achievement of the target stature in patients with CPP could be explained by the specific therapy for CPP to which the patients were subjected, and suggest that SO treatment does not negatively impact the outcome of CPP therapy.

A secondary aim of this study was the identification of prognostic factors for endocrine complications in patients with NT1. Our study confirms what was already observed by Ponziani et al. (11), where the frequency of overweight and obesity observed in CPP and patients with NT1 is almost identical to the frequency observed in patients with NT1 alone. Obesity alone does not appear to be mutually associated with this endocrine disorder. A predictor of CPP, instead, seems to be the age at the onset of NT1: in fact, the more the NT1 begins earlier the more the rate of CPP increases, suggesting that the age at which NT1 begins is intrinsically critical to these comorbidities (11). However, in the absence of a larger sample of very young (i.e. below the critical age for CPP diagnosis) we could not apply a multivariate regression model to demonstrate this aspect, calling for further data collection or multicenter studies. Another predictive factor could be the CSF-hypocretin levels at diagnosis, as reduced levels could be suggestive of CPP.

In conclusion, our results confirm the complexity of NT1 is not restricted to sleep-wake regulation but affects multiple endocrine aspects. In fact, in addition to sleep-related symptoms, we confirmed that patients with NT1 could present metabolic and endocrine complications (2). The high rate of misdiagnosis in these patients and their association with endocrine disorders as CPP, highlight the importance of the diagnosis of NT1 in the pediatric population. Our study also demonstrates for the first time that NT1 patients can achieve a final height in the range of the target height, a finding that was confirmed also in subgroups with different treatments (i.e. SO) and endocrinological comorbidities (i.e.CPP, overweight/obesity). All pediatric patients with a new NT1 diagnosis should be evaluated from an endocrinological point of view, especially prepubertal patients.

The strength of this study is the large sample of patients evaluated in a homogeneous way in two specialized centers of the third level and a long follow-up period. The limits instead are represented by the retrospective character of the study and the lack of a control group. Prospective clinical observations and randomized trials will be needed to increase the knowledge of NT1’s natural history, response to treatment, and severity. Future study perspectives including more patients with CPP will address a possible link between early-onset CPP and NT1-related signs and symptoms.

## Data availability statement

The original contributions presented in the study are included in the article/supplementary material. Further inquiries can be directed to the corresponding author.

## Ethics statement

The studies involving human participants were reviewed and approved by Comitato etico interaziendale Bologna-Imola (CE-BI). Written informed consent to participate in this study was provided by the participants’ legal guardian/next of kin.

## Author contributions

All authors provided contributions to study conception and design, acquisition of data, drafting the article or revising it critically for important intellectual content, and final approval of the version to be published. Clinical data from 72 patients in this study were reported in previous studies (9, 10). Here are the most important contributions of each author: SC, VA, FP study conception and design, acquisition of data, drafting the article; FP, AB, MG drafting the article and revising it critically; GP, AP, AC study conception and design, drafting the article and revising it critically. The study was designed, conducted, analyzed, and reported entirely by the authors.
